# Simultaneous detection of mRNA and protein stem cell markers in live cells

**DOI:** 10.1186/1472-6750-9-30

**Published:** 2009-04-02

**Authors:** Won Jong Rhee, Gang Bao

**Affiliations:** 1 Department of Biomedical Engineering, Georgia Institute of Technology and Emory University, Atlanta, Georgia 30332, USA

## Abstract

**Background:**

Biological studies and medical application of stem cells often require the isolation of stem cells from a mixed cell population, including the detection of cancer stem cells in tumor tissue, and isolation of induced pluripotent stem cells after eliciting the expression of specific genes in adult cells. Here we report the detection of Oct-4 mRNA and SSEA-1 protein in live carcinoma stem cells using respectively molecular beacon and dye-labeled antibody, aiming to establish a new method for stem cells detection and isolation.

**Results:**

Quantification of Oct-4 mRNA and protein in P19 mouse carcinoma stem cells using respectively RT-PCR and immunocytochemistry confirmed that their levels drastically decreased after differentiation. To visualize Oct-4 mRNA in live stem cells, molecular beacons were designed, synthesized and validated, and the detection specificity was confirmed using control studies. We found that the fluorescence signal from Oct-4-targeting molecular beacons provides a clear discrimination between undifferentiated and retinoic acid-induced differentiated cells. Using deconvolution fluorescence microscopy, Oct-4 mRNAs were found to reside on one side of the cytosol. We demonstrated that, using a combination of Oct-4 mRNA-targeting molecular beacon with SSEA-1 antibody in flow cytometric analysis, undifferentiated stem cells can be clearly distinguished from differentiated cells. We revealed that Oct-4 targeting molecular beacons do not seem to affect stem cell biology.

**Conclusion:**

Molecular beacons have the potential to provide a powerful tool for highly specific detection and isolation of stem cells, including cancer stem cells and induced pluripotent stem (iPS) cells without disturbing cell physiology. It is advantageous to perform simultaneous detection of intracellular (mRNA) and cell-surface (protein) stem cell markers in flow cytometric analysis, which may lead to high detection sensitivity and efficiency.

## Background

Embryonic stem cells (ESCs) have the potential to indefinitely self-renew and differentiate into any cell type [1], and extensive studies have been carried out to take advantage of these unique characteristics including tissue regeneration and repair [2-5]. In particular, recent advances in creating induced pluripotent stem (iPS) cells using human skin cells have opened a new avenue for the generation of stem cells without the use of embryos. Although embryonic stem cells provide an excellent source of cell lines for tissue repair and therapeutic applications, their embryonic origins can create ethical concerns and it is impossible to find cells that would have the identical genetics to match that of a patient. Therefore, it is very attractive to use iPS cells derived from a patient's adult cells to differentiate into specialized cells for treating specific diseases or repairing injured tissue. However, currently, the process of developing iPS cells is very inefficient – it was reported that only 10–20 iPS cell colonies were obtained from 0.1 million initial fibroblasts [6,7]. Therefore, it is necessary to have an efficient method to isolate iPS cells from mixed cell populations.

Another area of active research is cancer stem cells. In particular, embryonal carcinoma (EC) cells are pluripotent stem cells derived from teratocarcinomas and are considered the malignant counterparts of human embryonic stem (ES) cells [8,9]. EC cells can generate cancer cells after differentiation and form tumors after transplantation. Since cancer stem cells are known to reside in tumors, an effective therapy of cancer may require the specific detection and elimination of cancer stem cells in tumor [10,11]. Therefore, it is important to develop new technologies to effectively discriminate cancer stem cells from other cancer cells using specific gene and/or protein markers. It is also desirable to isolate cancer stem cells from tumor tissue for *in vitro *analysis of cancer stem cell biology.

Methods have been developed to isolate stem cells using antibodies that specifically bind to cell surface marker proteins [12-14], or based on transfection of plasmid with the promoter and reporter genes [15,16]. It is also possible to identify peptides that bind to surface markers of embryonal carcinoma cells (such as undifferentiated P19 cells) using a phage display library [17]. Although these methods can provide decent purity in isolating stem cells, each has limitations in its applicability. The strategy of targeting cell surface proteins in detecting stem cells relies on the available cell surface markers, which may be very limited; their expression levels may be too low for fluorescence-activated cell sorting (FACS) analysis. On the other hand, it is impossible to detect cancer stem cells by transfecting certain genes to cells *in vivo*, and the incorporation of foreign genes into stem cell chromosomes for cell isolation may cause concerns when these cells are used in treating human disease. Therefore, it is necessary to develop new methods for stem cell detection and isolation with better robustness and safety.

In this work, we have developed a new method for detection and isolation of stem cells by targeting both an intracellular marker (mRNA) and a cell surface marker (SSEA-1 protein), using mouse EC cells as a model system. Specifically, we designed molecular beacons to target Oct-4 mRNA in live EC cells in addition to targeting SSEA-1 protein using dye-labeled antibodies, and used flow cytometry to demonstrate the effectiveness in processing a large number of cells. Oct-4 is well-known key transcription factor regulating the differentiation and its down-regulation by siRNA induces differentiation of not only embryonic stem cells but also cancer stem cells [18,19]. Therefore, the level of Oct-4 mRNA in live stem cells can be used as an effective marker to indicate the state of stem cells (before and after differentiation). Oct-4 has also been implicated as a marker of germ cell tumors. Here we demonstrate that, by imaging the level of Oct-4 mRNA using molecular beacons, undifferentiated normal and cancer stem cells can be distinguished from other cells.

## Results

### Decrease in Oct-4 mRNA and protein levels in EC cells after differentiation

Retinoic acid (RA) treatment has been widely used to regulate gene expression. For example, DNA microarray analysis of ES-D3 cells showed the down-regulation of 18 genes and the up-regulation of 61 genes after RA treatment [20]. RA treatment can also regulate many genes [21] in EC cells, including Oct-4, and induce cell differentiation to different cell types including neurons, glial cells and fibroblasts, depending on RA concentration [22,23]. Extensive studies have indicated that Oct-4 is down-regulated as stem cells start to differentiate.

**Figure 1:**
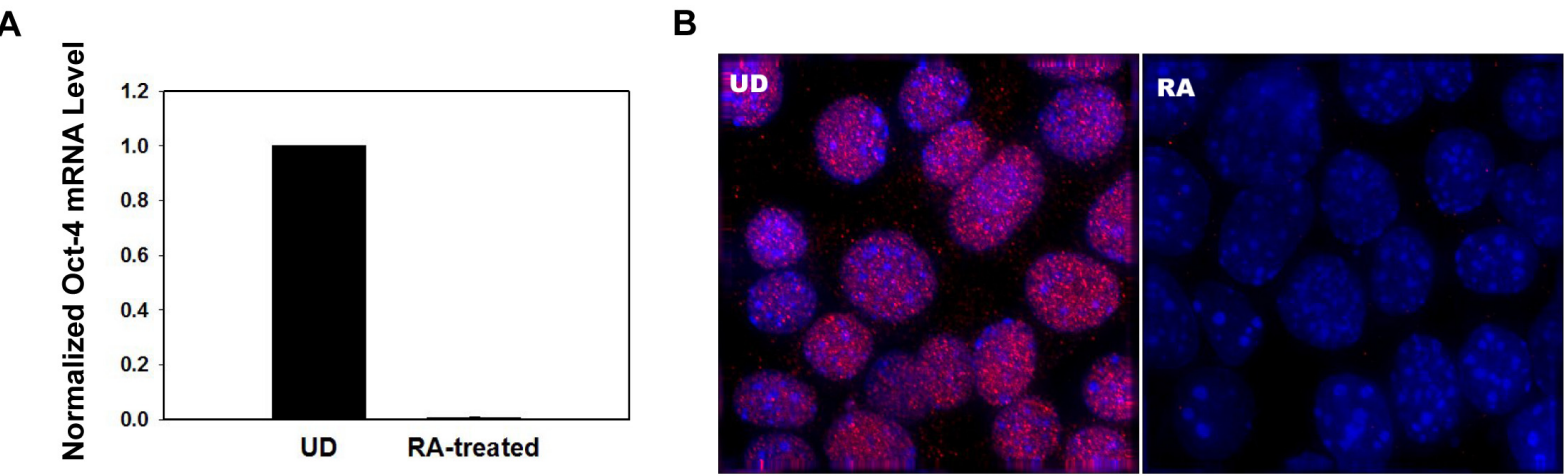
**Changes of Oct-4 mRNA and protein levels 4 days after retinoic acid (RA) treatment in P19 embryonal carcinoma cells**. **A**. Real-time PCR result of Oct-4 mRNA level in undifferentiated (UD) and differentiated (RA treated) P19 cells. **B**. Immunocytochemistry of Oct-4 protein (Red) before (UD) and after (RA) differentiation. Cell nuclei were stained with Hoechst 33342 (Blue).

As a positive control for molecular beacon based studies, we have quantified the changes in Oct-4 mRNA level 4 days after RA treatment in P19 cells. Cells were exposed to 500 nM of RA for 2 days followed by 2 days of incubation with medium absent of RA. As shown in Figure 1A, our real-time PCR results indicate that RA treatment decreased the Oct-4 mRNA level in differentiated cells to less than 1% of that in untreated EC cells. To check if decrease in Oct-4 mRNA level reflects changes in Oct-4 protein level, we carried out immunocytochemistry assays of Oct-4 protein before and after RA treatment using dye-labeled antibody for Oct-4. As demonstrated in Figure 1B, strong fluorescence signals were observed in the nuclei of undifferentiated cells, while there was essentially no signal after RA treatment. Since Oct-4 is a transcription factor, it is not surprising that the staining of Oct-4 protein is predominately in cell nucleus (Figure 1B).

### Design and validation of Oct-4 targeting molecular beacons

We have designed molecular beacons to image changes in Oct-4 mRNA level in live EC cells before and after differentiation. Molecular beacons are dual-labeled antisense oligonucleotide (ODN) probes with a fluorophore and a quencher at each ends [24,25]. In the absence of a complementary target, they form a stem-loop hairpin structure, and the fluorescence reporter (fluorophore) is quenched by the quencher. Hybridization with the target mRNA opens the stem-loop structure and physically separates the reporter from quencher, allowing a fluorescence signal to be emitted upon excitation.

**Figure 2:**
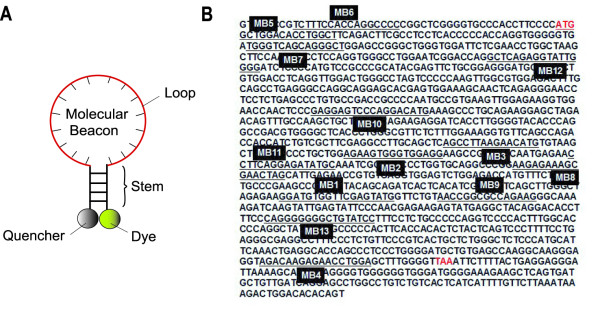
**The cDNA sequence of mouse Oct-4 mRNA and the hybridization sites of Oct-4 targeting molecular beacons (MB1 – MB13)**.

To ensure that there is good target accessibility in live-cell detection of Oct-4 mRNA, we have designed and tested 13 molecular beacons (designated as MB1 to MB13) that target different sites on Oct-4 mRNA, as shown in Figure 2. Target accessibility is one of the most important issues in live-cell mRNA detection since, although a molecular beacon can be designed to have its probe sequence unique to the target mRNA, the target sequence may not necessarily be accessible in a living cell. Specifically, the probes need to avoid targeting sequences that form secondary structures or are occupied by RNA-binding proteins [26] in order to have a good level off signal.

As shown in Figure 2 and Table 1, thirteen molecular beacons were designed to target different sites in Oct-4 mRNA, including siRNA binding sites (MB1, MB8, MB12), the anti-sense oligonucleotide binding sites (MB2) predicted using mFOLD , loop site predicted by mFOLD  (MB3), the sites close to termination codon (MB4) and translation initiation codon (MB5), respectively, 5' untranslated region (MB6), and exon-exon junctions (MB1, MB10, MB11). The target sequences for MB7, MB9, and MB13 were chosen without any particular reason. As a negative control, we used a random-sequence molecular beacon (random beacon) which does not have any complementary target in human and mouse genome. All molecular beacons have Cy3 as the fluorophore and BHQ2 as the quencher. After synthesis, these molecular beacons were tested in solution to ensure that they have an acceptable signal-to-background ratio (> 5).

**Table 1:** The design of mouse Oct-4 targeting and negative control (random) molecular beacons*

**Beacon ID**	**Molecular Beacon Design**
MB1	5'-Cy3-CGTCGCATACTCGAACCACATCCCGACG-BHQ2-3'
MB2	5'-Cy3-CAGCCCCTCCACCCACTTCTGGCTG-BHQ2-3'
MB3	5'-Cy3-CCGGTCATGTTCTTAAGGCTACCGG-BHQ2-3'
MB4	5'-Cy3-CGCAGTCCAGGTTCTCTTGTCTCTGCG-BHQ2-3'
MB5	5'-Cy3-CGGAGAGCCAGGTGTCCAGCCATCTCCG-BHQ2-3'
MB6	5'-Cy3-CCGGAGGGGCCTGGTGGAAAGATCCGG-BHQ2-3'
MB7	5'-Cy3-CCACAGAGCCCTGCTGACCCACTGTGG-BHQ2-3'
MB8	5'-Cy3-CAGGACCTAGTTCGCTTTCTCTTGTCCTG-BHQ2-3'
MB9	5'-Cy3-CGCAGCTTCTGGCGCCGGTTCTGCG-BHQ2-3'
MB10	5'-Cy3-CCGGAACATGTCCTGGGACTCCTCTTCCGG-BHQ2-3'
MB11	5'-Cy3-CGAGCGCATATCTCCTGAAGGGCTCG-BHQ2-3'
MB12	5'-Cy3-CGTGCCCCCAATACCTCTGAGCGCACG-BHQ2-3'
MB13	5'-Cy3-CCGTGGGATACAGCCCCCCCTGCACGG-BHQ2-3'
Random MB	5'-Cy3-CGACGCGACAAGCGCACCGATACGTCG-BHQ2-3'

* The underline indicates the stem sequence. Abbreviation: MB, molecular beacon; BHQ, black hole quencher.

### Detection of Oct-4 mRNA in undifferentiated and differentiated EC Cells

**Figure 3:**
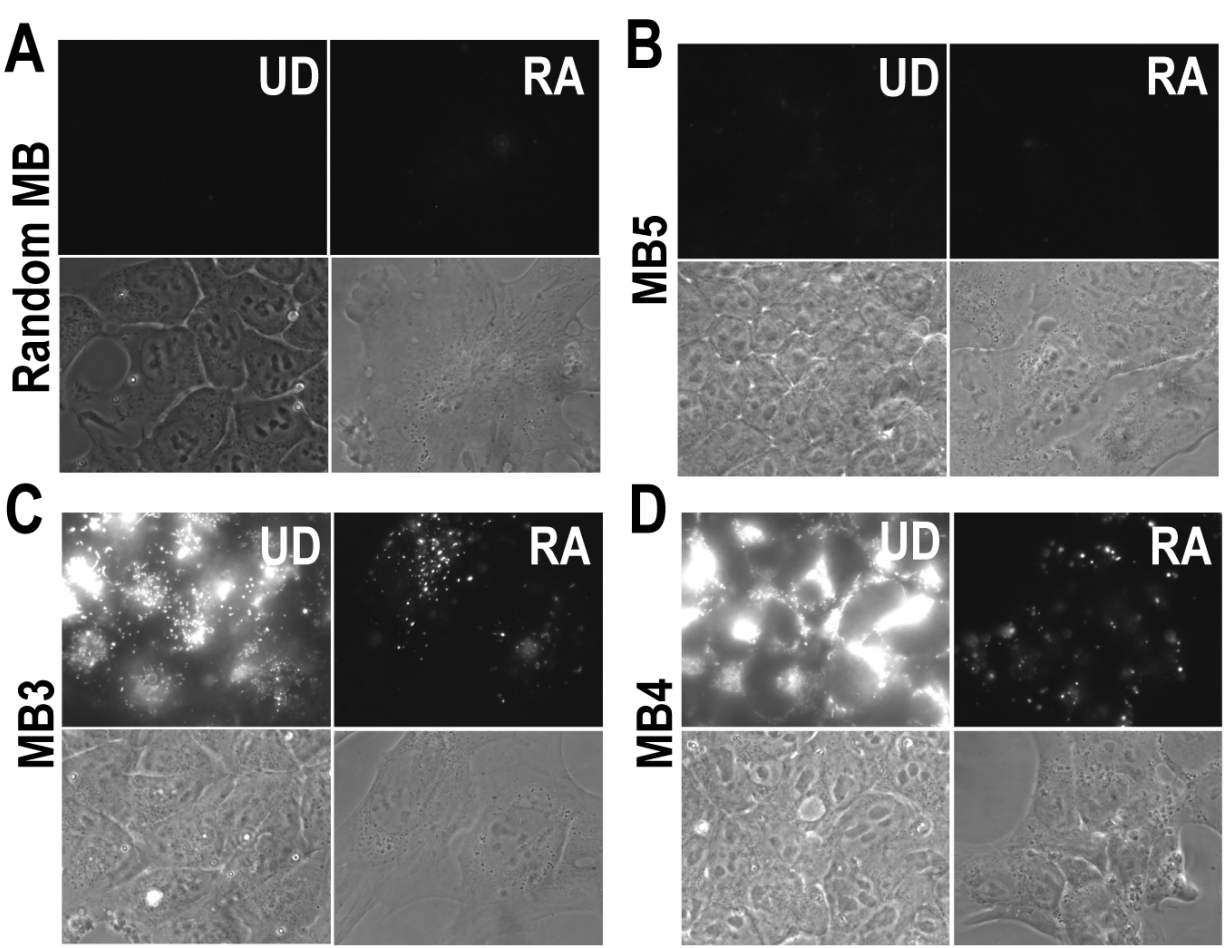
**Fluorescence signal from different molecular beacon designs**. In A-D, left and right panels display respectively the undifferentiated (UD) and RA treated (differentiated) cells; top and bottom panels display respectively the epifluorescence images of beacons and the bright field images of the same cells. The same exposure time was used for all beacon fluorescence imaging. **A **and **B**. Random beacon used as negative control (A) and MB5 (B) gave very low signal. Signals from MB1, MB6-MB13 were similar to that of MB5. **C **and **D**. MB3 (C) and MB4 (D) gave high signal levels, indicating good target accessibility.

It is well known that RA treatment induces P19 cells to differentiate into glial, neuron and fibroblast-like cells [22], and the Oct-4 expression level decreases significantly after differentiation (Figure 1). We delivered each of the thirteen beacons synthesized into both undifferentiated and differentiated cells by streptolysin O (SLO) and analyzed with epi-fluorescence microscope. We could only observe very low fluorescence signal from Oct-4 targeting molecular beacons MB1, MB5 – MB13 in undifferentiated cells, as shown in Figure 3A (representative images), and the random beacons used as a negative control did not give much fluorescence signal in undifferentiated cells (Figure 3B). We believe that the sites on Oct-4 mRNA targeted by MB1, MB5-MB13 are not accessible for molecular beacons to hybridize due to either the secondary structures formed at, or the RNA-binding proteins bound to these regions. However, MB2, MB3 and MB4 all showed strong fluorescence signal in undifferentiated cells, and much lower fluorescence signal in differentiated cells, as demonstrated by Figures 3C and 3D, respectively. Flow cytometric analysis of hybridization of molecular beacons to Oct-4 mRNA confirmed that only MB2, MB3 and MB4 gave shifts of fluorescence signal distribution in undifferentiated cells compared with that in differentiated cells or random beacon signal distribution in undifferentiated cells (data not shown). We found that MB4 gave the highest signal intensity in undifferentiated cells and therefore, in the subsequent experiments, only MB4 was used.

### Deconvolution microscopy of Oct-4 mRNA

**Figure 4:**
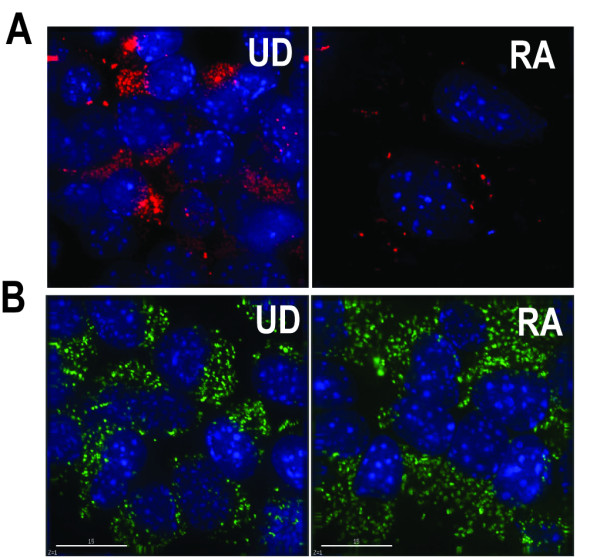
**Deconvolution fluorescence imaging of Oct-4 mRNA and total mRNA in live P19 cells, with cell nucleus stained with Hoechst 33342 (blue)**. **A**. The cytosolic localization of Oct-4 mRNA (red) in undifferentiated (UD) and RA treated (differentiated) cells. **B**. Total RNA staining using SytoRNA Select dye (green) in undifferentiated (UD) and RA treated (differentiated) cells. Scale bar = 15 μm.

We used deconvolution microscope to image and analyze the localization of Oct-4 mRNA. As shown by the top panel in Figure 4A, in most of the undifferentiated cell, beacon signals (red) from detecting Oct-4 mRNA are mostly localized on one side of the cell cytosol, and the fluorescence signal intensity varied from cell to cell, indicating the differences of Oct-4 expression level among cells. As demonstrated previously, molecular beacon signal from targeting Oct-4 mRNA in differentiated cells was very low, confirming that the beacon signal was specific. We have also fluorescently labeled total RNA using SytoRNA Select in both undifferentiated and differentiated live cells. As shown in Figure 4B, it appears that there was little difference in total RNA staining pattern in undifferentiated and differentiated cells. This further confirms the Oct-4 mRNA detection specificity using molecular beacons.

### Co-labeling of SSEA-1 protein and Oct-4 mRNA

**Figure 5:**
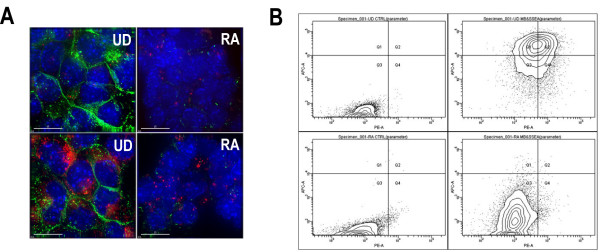
**Simultaneous targeting of Oct-4 mRNA and SSEA-1 protein in live P19 cells analyzed by deconvolution microscopy and flow cytometry**. **A**. Deconvolution microscopy imaging of Oct-4 mRNA and SSEA-1 protein. Top panel shows that targeting of SSEA-1 protein using dye-labeled antibody gave strong signal (green) in undifferentiated (UD) cells and very weak signal in RA-treated (differentiated) cells. Random beacons only gave very weak signal (red). Bottom panel shows the fluorescence images of simultaneous targeting of Oct-4 mRNA (red) and SSEA-1 protein (green) in undifferentiated (UD) RA-treated (RA) cells. Scale bar = 15 μM. **B**. Flow cytometric analysis of signal from targeting Oct-4 mRNA and SSEA-1 protein in undifferentiated (top panel) and differentiated (bottom panel) cells. Right and left panels show respectively the results of cells with and without MB and SSEA-1 antibody. X- and Y-axis show respectively the relative fluorescence intensities of imaging Oct-4 mRNA and SSEA-1 protein.

It has been well established that undifferentiated EC cells express a stage-specific embryonic antigen, SSEA-1 protein on their surface. We therefore imaged SSEA-1 protein using dye-labeled antibody in addition to targeting Oct-4 mRNA in live cancer stem cells to see if we could use both signals in a flow cytometry analysis. SSEA-1 antibodies conjugated with Alexa 647 were incubated with cells after molecular beacon delivery by SLO. The fluorescence signals were then analyzed using a deconvolution microscope. Shown in Figure 5A were images of fluorescence signal from targeting Oct-4 mRNA (red) and SSEA-1 protein (green) in live undifferentiated and differentiated (RA-treated) P19 cells. The top panel of Figure 5A demonstrates that signal (green) from SSEA-1 staining is quite high in undifferentiated cells and is very low in differentiated cells. As a negative control, random beacons were delivered into cells before and after differentiation and showed very weak signal in both cell types (red signal in the top panel), in contrast to the SSEA-1 signal. With Oct-4 targeting molecular beacons (MB4), strong fluorescence signal (red) was detected in undifferentiated cell, together with strong signal from SSEA-1 protein (lower panel in Figure 5A), and only low beacon signal (red) can be detected in differentiated cells, along with very low (green) signal from SSEA-1. These imaging results show clearly that signals from Oct-4 targeting beacons and SSEA-1 stating using antibodies are highly correlated and therefore could be used in detecting cancer stem cells with higher sensitivity than using one marker only.

Flow cytometric analysis of SSEA-1 protein and Oct-4 mRNA expression in undifferentiated and differentiated P19 cells using dye-labeled antibody and molecular beacons, respectively, was performed. The rational is that only a very small number of cancer stem cells (e.g., 1 in 1,000,000 cells) in tumor tissue, and therefore, in detecting cancer stem cells, flow cytometry is likely the most efficient method. In the flow cytometry assay, both undifferentiated and RA treated cells were detached, and 1 μM of Oct-4 targeting molecular beacons was delivered by SLO in suspension. Alexa 647 dye labeled SSEA-1 antibody was incubated after beacon delivery in serum-containing medium for 30 min. Cells without Oct-4 antibody and molecular beacon were used as control. As demonstrated in Figure 5B, left panel, flow cytometry analysis of cells without any probe showed no much difference in terms of auto-fluorescence in both undifferentiated (left top panel) and differentiated (left bottom panel) cells. In Figure 5B, X-axis shows the relative fluorescence intensity from Oct-4 mRNA and Y-axis shows the relative fluorescence intensity from dye-labeled SSEA-1 antibody. For undifferentiated cells (right top panel), fluorescence signals from SSEA-1 protein (Y-axis) and Oct-4 mRNA (X-axis) were significantly shifted compared to control cells (left panel). In contrast, there were only relatively small shifts of fluorescence signal from both SSEA-1 protein and Oct-4 mRNA fluorescent signals in differentiated cells (right bottom panel). The results shown in Figure 5B clearly demonstrate that, using flow cytometry assays in combination with two stem cell markers (Oct-4 mRNA and SSEA-1 protein), cancer stem cells could be detected from other tumor cells.

## Discussion

Although cell surface markers can be used to detect stem cells, medical applications of stem cell technology, including the detection of iPS cells from a heterogeneous cell population and the isolation of cancer stem cells from tumor tissue often requires the specific targeting of intracellular stem cell markers, such as mRNAs. However, to our knowledge, the detection of stem cell mRNA marker in living cells has not been demonstrated. Here we report the detection of Oct-4 mRNA and SSEA-1 protein in live carcinoma stem cells using respectively molecular beacons and dye-labeled antibody, aiming to establish a new method for stem cells detection and isolation. Although in this work we used mouse embryonal carcinoma cells as a model, the same approach is applicable in detecting iPS and cancer stem cells. Live-cell detection of stem cell marker mRNAs is also of importance in tracking embryonic stem cell differentiation and the isolation of other stem cells such as progenitor cells by detecting specific mRNAs that can discriminate target cells from other cells.

There are three important issues in detecting live stem cells (including iPS cells and cancer stem cells) from heterogeneous cell population or tissue: detection specificity, sensitivity, and efficiency. Although cell surface protein markers can be used for detecting live stem cells, they have very limited availability. Therefore, the detection of stem cell marker mRNAs using molecular beacons is attractive in that there are many mRNAs available for targeting and the beacon based mRNA detection does not require cells to be fixed or lysed. Clearly, detecting stem cells using multiple mRNA markers in combination with surface protein markers could increase both detection specificity and sensitivity. Detection efficiency is also an important issue, since the success rate of getting iPS cells is rather low: typically less than 0.01% of all cells transfected are converted into iPS cells [27]. Further, cancer stem cells are very rare, estimated as 1 in 100,000 to 1,000,000 cancer cells in tumor tissue. Therefore, the use of a flow cytometry method may be necessary to process a large number of cells in detecting iPS cells or cancer stem cells.

For sensitive isolation of iPS cells, it may be necessary to detect multiple stem cell markers. For example, concerting skin cells to iPS cells requires specific expression of transcription factors Oct-4, Sox2 and Nanog, as demonstrated recently [6,7]. It is likely that the expression of these genes has different levels in different cells when inducing them into iPS. Therefore, it may be advantageous to design molecular beacons to target mRNAs corresponding to multiple factors, since it is fairly easy to simultaneously use 3–5 molecular beacons either with the same fluorophore (to enhance signal) or with different fluorophores (to distinguish different mRNAs). The molecular beacon based method is likely to be much better than the antibody-based method in that the availability of target molecules (mRNAs) is quite large and the cost of synthesizing molecular beacons is low compared with that of generating antibodies (even if multiple cell surface protein markers are available). The efficiency and specificity of stem cell detection and isolation can be further enhanced by targeting mRNAs using multiple molecular beacons and cell surface markers using antibodies.

For detecting cancer stem cells inside a solid tumor using tumor tissue, it may be sufficient to target stem cell markers such as Oct-4 and SSEA-1 in order to positively identify them, since these markers are highly expressed only in stem cells, and the tumor type is known *in priory*. However, when using other samples such as blood, it will be necessary to add a marker that is highly expressed only in specific cancer stem cells so that normal stem cells would not be taken as cancer stem cells. Identifying such a marker for the specific tumor type is an important research topic.

**Figure 6:**
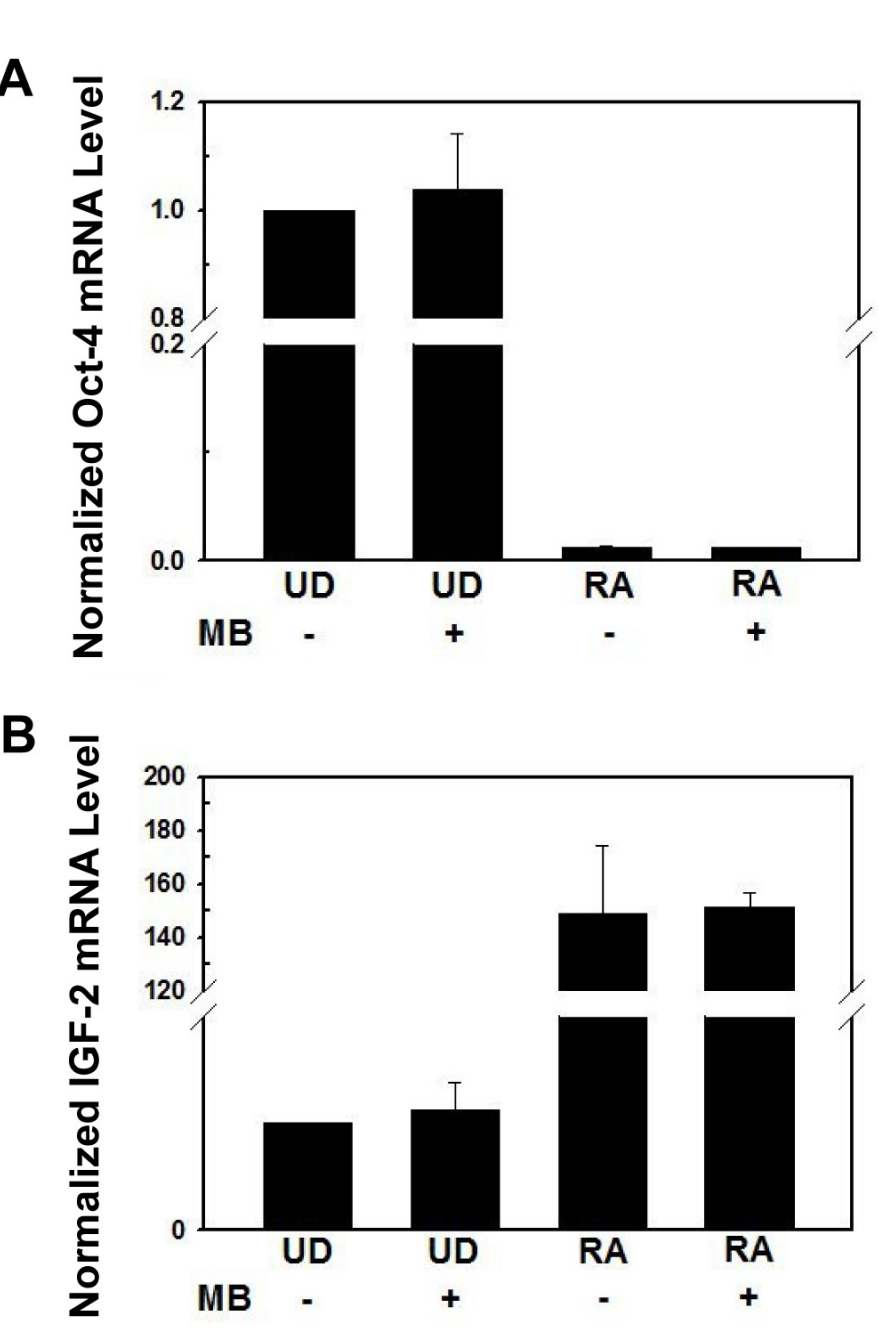
**The effect of Oct-4 targeting molecular beacon (MB4) on Oct-4 and IGF-2 mRNAs before and after differentiation**. Shown in A and B are respectively the relative amount of Oct-4 and IGF-2 mRNAs in undifferentiated (UD) and RA-treated (RA) cells with (MB+) and without (MB-) Oct-4 targeting beacon MB4.

In this work, we have demonstrated the use of molecular beacons to detect Oct-4 mRNA as a new method for the detection of cancer stem cells among normal cells or cancer cells. A particular issue is whether the biology of cancer stem cells is affected by the detection method, including the delivery approach and the probes inside the living cell. It is unlikely that unbound molecular beacon itself would affect cell biology such as gene expression and cell differentiation, since the oligonucleotide, fluorophore and quencher are not toxic. The effect of the delivery method using streptolysin *O *(SLO), however, needs to be determined, since SLO binds to cholesterol molecules on cell plasma membrane which may cause damages to cell membrane [28,29]. To address this issue, we delivered 1 μM of MB4 into undifferentiated P19 cells by SLO, and cultured these cells for 2 weeks. Further, undifferentiated cells with and without MB4 were cultured for 10 days and then treated with RA for 4 days (totally 2 weeks). The mRNA levels of Oct-4 and IGF-2 (Insulin-like Growth Factor-2) in these cells measured using RT-PCR are shown in Figure 6. Evidently, cells with and without MB4 had the same expression levels of Oct-4 (left panel) and IGF-2 (right panel) before and after differentiation, suggesting that SLO-based beacon delivery and probe/target hybridization did not have significant effects on cell physiology including gene expression, self-renewal, and differentiation. We also delivered Oct-4 targeting molecular beacons by SLO into mouse ESC, ES-D3 cells, for the isolation of ESCs from their differentiated cells. RT-PCR results and cell morphologies of differentiated cells with and without beacon indicated that there was no change in the ability of stem cell differentiation (unpublished data). To further establish the molecular beacon based method for detection and sorting of stem cells (including cancer stem cells and iPS cells), research is being conducted to demonstrate the sensitivity, specificity and safety of this technique. The results will be reported in a subsequent publication.

## Conclusion

In this work we have established a novel method for detecting and isolating stem cells by simultaneously targeting stem cell specific mRNA and protein markers. We found that molecular beacons can be designed to fluorescently image Oct-4 mRNA in cell cytoplasm with high specificity without disturbing cell physiology. Although there exist cell surface protein markers for stem cells, their availability is very limited and therefore, it is very appealing to detect intracellular stem cell markers such as mRNAs. We also found that, by performing simultaneous detection of Oct-4 mRNA using molecular beacons and SSEA-1 protein on cell surface in a flow cytometric assay, the detection sensitivity and efficiency can be enhanced. Multiple beacons can be using to image different mRNAs to further increase signal level and achieve better specificity. Taken together, we demonstrated that the detection of specific mRNAs as stem cell markers using molecular beacons, in combination with targeting cell surface markers in a flow cytometric analysis has the potential to provide a powerful tool for stem cell research, including the detection of cancer stem cells and the isolation of iPS cells.

## Methods

### Cell culture

Mouse EC cells, P19, were purchased from ATCC (CRL-1825; American Type Culture Collection) and grown in α-minimum essential medium (Gibco BRL) containing 7.5% calf bovine serum and 2.5% of fetal bovine serum (Gibco BRL). For differentiation, cells were transferred to 60 mm culture dish with 500 nM all-trans retinoic acid (RA) (Sigma). 2 days after RA treatment, cells were transferred without RA and further cultured for 2 days.

### Real-time quantitative PCR

Total RNA was isolated from cells using Qiagen RNeasy Mini Kit. 100 ng of total RNA was used for cDNA synthesis by random hexamers with Invitrogen Thermoscript RT-PCR kit. For real-time PCR, the cDNA was amplified using a Stratagene Mx3005P (Stratagene) RT-PCR machine. The Ambion's 18S primers were used as an internal control for real-time PCR. PCR amplification was performed with the following primers with 60°C as the annealing temperature: Oct-4 sense: 5'-CCGTGTGAGGTGGAGTCTGGAG-3' and Oct-4 anti-sense: 5'-GCGATGTGAGTGATCTGCTGTAGG-3', IGF-2 sense: 5'-GTCGATGTTGGTGCTTCTCATCTC-3' and IGF-2 anti-sense: 5'-GAAGCAGCACTCTTCCACGATG-3'. The Oct-4 mRNA levels were normalized against the 18S rRNA levels.

### Molecular beacon synthesis, delivery and fluorescence imaging

Molecular beacons with DNA (2'-deoxy) backbone labeled with Cy3 fluorophore at the 5' end and Black Hole quencher 2 (BHQ2) at the 3' end were synthesized by MWG Biotech (High Point, North Carolina). Molecular beacons were delivered into live P19 cells cultured in 4-well chamber with a reversible permeabilization method using activated SLO [30,31] at the concentration of 0.2 U/mL in serum free medium for 15 min. After delivery, the SLO containing medium was changed with the normal growth medium and incubated for 30 min before fluorescence imaging. The fluorescence imaging of live cells was performed using a Zeiss Axiovert 100 TV epifluorescence microscope coupled to a Cooke Sensicam SVGA cooled CCD camera. The confocal imaging was done with Zeiss Axiovert LSM-100 confocal microscope. The Cy3-labeled beacons were visualized with excitation at 545 nm and emission detection at 570 nm. Deconvolution microscopic analysis was carried out using DeltaVision Deconvolution Microscope (Applied Precision, INC.). The sequences of all beacons designed and tested are shown in Table 1, with the underlined sequences as stem sequences. The numbers were assigned by the order of test for target specificity. The target sequences of Oct-4 mRNA are also illustrated in Figure 2. Each molecular beacon experiment was performed at least 3 times to ensure reproducibility.

### Immunocytochemistry analysis

For Oct-4 protein staining, cells were fixed with 4% paraformaldehyde for 15 min followed by permeabilization with 0.2% Triton X-100. Cells were blocked with 5% goat serum in PBS for 30 min at 37°C and incubated with blocking solution containing mouse monoclonal Oct-4 antibody (Santa Cruz Biotechnology, INC.) for 30 min at 37°C. After washing 3 times, cells were then incubated with secondary mouse antibody conjugated with Alexa Fluor 550 (Molecular Probes) for 30 min at 37°C.

### Flow cytometric analysis of Oct-4 mRNA and SSEA-1 protein

P19 cells were detached by Trypsin-EDTA or Enzyme-free cell dissociation buffer (Invitrogen) and washed once with serum-free medium. 1 μM of molecular beacons was delivered using 2 U/mL SLO in serum-free medium for 15 min. After washing, cells were incubated with SSEA-1 antibody (1:50, Santa Cruz Biotechnology, INC) for 30 min, and washed twice and then filtered to obtain single cell population. Analysis was carried out using FACS Diva (Beckman Coulter, INC).

## Abbreviations

iPS cell: induced pluripotent stem cell; EC cell: embryonal carcinoma cell; ES cell: embryonic stem cell; RA: retinoic acid; MB: molecular beacon; SLO: streptolysin O; IGF-2: Insulin-like Growth Factor-2.

## Authors' contributions

WJR conducted the experiments and analyzed the results including cell culture, RT-PCR, immunocytochemistry, design of molecular beacons, and molecular beacon and SSEA-1 protein imaging in live cells. GB financially supported the research and analyzed and interpreted the data. Both authors conceived of the study, and participated in its design and coordination, and manuscript writing.
